# Specific targeting of PDGFRβ in the stroma inhibits growth and angiogenesis in tumors with high PDGF-BB expression

**DOI:** 10.7150/thno.37851

**Published:** 2020-01-01

**Authors:** Maria Tsioumpekou, Sara I. Cunha, Haisha Ma, Aive Åhgren, Jessica Cedervall, Anna-Karin Olsson, Carl-Henrik Heldin, Johan Lennartsson

**Affiliations:** 1 Department of Medical Biochemistry and Microbiology, Uppsala University, Sweden.; 2 Department of Pharmaceutical Biosciences, Uppsala University, Sweden.; 3 Ludwig Institute for Cancer Research, Uppsala Branch, Uppsala University, Sweden.; 4 Department of Immunology, Genetics and Pathology, Uppsala University, Sweden.; 5 Department of Neuroscience, Uppsala University, Sweden.

**Keywords:** Low molecular weight inhibitor, PDGFRβ, pericytes, tumor growth, angiogenesis

## Abstract

PDGF-BB/PDGFRβ signaling plays an important role during vascularization by mediating pericyte recruitment to the vasculature, promoting the integrity and function of vessels. Until now it has not been possible to assess the specific role of PDGFRβ signaling in tumor progression and angiogenesis due to lack of appropriate animal models and molecular tools.

**Methods:** In the present study, we used a transgenic knock-in mouse strain carrying a silent mutation in the PDGFRβ ATP binding site that allows specific targeting of PDGFRβ using the compound 1-NaPP1. To evaluate the impact of selective PDGFRβ inhibition of stromal cells on tumor growth we investigated four tumor cell lines with no or low PDGFRβ expression, *i.e*. Lewis lung carcinoma (LLC), EO771 breast carcinoma, B16 melanoma and a version of B16 that had been engineered to overexpress PDGF-BB (B16/PDGF-BB).

**Results**: We found that specific impairment of PDGFRβ kinase activity by 1-NaPP1 treatment efficiently suppressed growth in tumors with high expression of PDGF-BB, *i.e.* LLC and B16/PDGF-BB, while the clinically used PDGFRβ kinase inhibitor imatinib did not suppress tumor growth. Notably, tumors with low levels of PDGF-BB, *i.e.* EO771 and B16, neither responded to 1-NaPP1 nor to imatinib treatment. Inhibition of PDGFRβ by either drug impaired tumor vascularization and also affected pericyte coverage; however, specific targeting of PDGFRβ by 1-NaPP1 resulted in a more pronounced decrease in vessel function with increased vessel apoptosis in high PDGF-BB expressing tumors, compared to treatment with imatinib. *In vitro* analysis of PDGFRβ ASKA mouse embryo fibroblasts and the mesenchymal progenitor cell line 10T1/2 revealed that PDGF-BB induced NG2 expression, consistent with the *in vivo* data.

**Conclusion**: Specific targeting of PDGFRβ signaling significantly inhibits tumor progression and angiogenesis depending on PDGF-BB expression. Our data suggest that targeting PDGFRβ in the tumor stroma could have therapeutic value in patients with high tumor PDGF-BB expression.

## Introduction

The last few decades have witnessed an explosive development of small molecule inhibitors of receptor tyrosine kinases for targeted cancer therapy. It has also become clear that the tumor microenvironment, including infiltrating immune cells, cancer-associated fibroblasts, endothelial cells and pericytes of the tumor vasculature, play important roles for tumor growth, invasion and metastasis 1-4.

Induction of angiogenesis is a pivotal step in progression of solid tumors and therefore targeted therapy against the vascular compartment is an attractive strategy in anti-cancer treatment. Pericytes are smooth muscle-like cells, which are closely wrapped around capillary endothelial cells and have a key role in vascular development, stabilization, maturation and remodeling 5. The function and homeostasis of pericytes are regulated to a large extent by the platelet-derived growth factor (PDGF)/PDGF β-receptor (PDGFRβ) signaling pathway 6-9. Mice genetically deficient of PDGF-B or PDGFRβ display abnormal development and maturation of the vascular tree due to inefficient recruitment of pericytes and smooth muscle cells 5,10, which leads to lethality around the time of birth.

PDGF is a family of mitogens that stimulate, for example, the division of smooth muscle cells, pericytes, fibroblasts and glial cells in the brain. The PDGF isoforms (PDGF-AA, -BB, -AB, -CC and -DD) signal through activation of two structurally related receptor tyrosine kinases, PDGFRα and PDGFRβ 11-13. The different receptors bind the ligands with different affinities. PDGFRα binds preferentially PDGF-A, -B and -C, whereas PDGFRβ binds PDGF-B and -D 14. Activated PDGFRα and β subsequently trigger overlapping but also unique signaling outcomes 15.

Under physiological conditions, PDGF signaling regulates embryonic development 16*,* wound healing 17, interstitial fluid pressure 18, and the integrity of the blood brain barrier 19. On the other hand, overactive PDGF signaling has been observed in certain pathological conditions, including atherosclerosis, various fibrotic conditions and malignancies 20*.* Autocrine PDGF-BB signaling promotes growth of the skin tumor dermatofibrosarcoma protuberans (DFSP), and mutations of PDGF receptors drive certain gastrointestinal stromal tumors (GIST), hypereosinophilic syndrome and gliomas 21. Paracrine stimulations involving PDGF isoforms also play an important role in the development of stromal cancer-associated fibroblasts and promotion of tumor vascularization by stimulation of vascular smooth muscle cells or pericytes 20,22,23*.*

The prototypical PDGFRβ kinase inhibitor, imatinib (also named Glivec/Gleevec or STI571), is an ATP-competitive agent which is used clinically for the treatment of chronic myeloid leukemia (CML), GIST, DFSP as well as other tumor types 13,20,24*.* In addition to targeting the activity of the PDGFRs, imatinib also inhibits the kinase activities of c-Kit, Abl/Bcr-Abl and CSF1R; other registered PDGFR kinase inhibitors, such as sunitinib and sorafenib are even less selective 25. Overactive PDGF signaling has also been reported to be involved in various other tumor types and efforts have been made to target PDGFRs using imatinib, sorafenib or sunitinib among others 25,26. The multi-targeting characteristic of the available inhibitors, makes it difficult to uncover the specific importance of PDGFRβ in tumorigenesis, since the observed effects may be due to inhibition of other kinase targets.

Selective targeting of host kinases can be elegantly achieved by analogue-sensitive kinase allele (ASKA) technology, where the wild-type kinase is replaced by a kinase that is mutated in the ATP-binding pocket so that it can be specifically inhibited by a compound (1-NaPP1) that interferes uniquely with the ASKA mutant and does not inhibit other kinases. Animals bearing this silent mutation carry an otherwise fully functional kinase 27-29*.*

To determine the specific role of PDGFRβ in the tumor microenvironment, we have studied four tumor cell lines, with no or low PDGFRβ expression. These cell lines were grown in an ASKA mouse model with a mutant PDGFRβ kinase (Taconic Artemis). We have compared the effect of selective PDGFRβ kinase inhibition with the effects of the broader kinase inhibitor imatinib.

## Material and methods

### PDGFRβ ASKA mice and animal experimentation

PDGFRβ ASKA C57BL/6 mutant mice were kindly provided by Taconic Artemis and all animal experiments described were approved by the local committees for animal care and experimentation.

Eight to twelve weeks old PDGFRβ ASKA mice received subcutaneous inoculation of 1×10^6^ B16, B16/PDGF-BB or LLC cells in the left dorsal skinfold. EO771 metastatic breast cancer cells (5×10^5^) were orthotopically injected into the 4^th^ left mammary fat pad of female mice. When the tumors became palpable, the mice were randomized into different treatment groups to receive 150 μL oral gavage of either vehicle (sterilized phosphate-buffered saline (PBS)) or imatinib (75, 150 or 250 mg/kg/day) for 10 days, or intraperitoneal injection (100 μL) of vehicle (5% ethanol, 70% PEG400, 25% saline) or 1-NaPP1 (15, 30 or 45 mg/kg/day) for 10 days. The tumors were measured using calipers, and the tumor volume was calculated using the formula π/6 × length × width × width. All experiments were terminated whenever one of the experimental groups reached the ethically approved experimental endpoint of 1000 mm^3^ tumor volume or the day after treatment was finished. The mice were anesthetized with Avertin (Sigma) and perfused with 10 mL PBS followed by 10 mL 4% paraformaldehyde (PFA). Tumors were harvested and kept in 30% sucrose for 24 h, embedded in OCT cryopreservation medium (Bio-Optica), or kept in 4% PFA followed by 70% ethanol for paraffin embedding. No animals were excluded from the analysis.

### FITC-lectin perfusion assay

FITC-lectin perfusion was performed as described previously 30. The degree of perfusion was determined by the ratio FITC-lectin-positive area/CD31-positive area, analyzed automatically with CellProfiler (www.cellprofiler.org; 31), revealing the proportion of perfused vessels with functional blood flow.

### Isolation of mouse embryonic fibroblasts (MEFs) from PDGFRβ ASKA mice

Pregnant PDGFRβ ASKA mice were sacrificed at day 13 or 14 postcoitum by cervical dislocation and mouse embryonic fibroblasts was isolated as described elsewhere 32. After two days in culture, when the cells were 80-90% confluent, they were harvested, and frozen for future use.

### Cell culture

MEFs from both wild-type and ASKA PDGFRβ mutants, as well as the 10T1/2 mouse embryonic cell line, MOVAS mouse smooth muscle cells, BJ foreskin human fibroblasts, Lewis Lung Carcinoma (LLC) cell line, EO771 metastatic breast cancer cell line, B16 and B16/PDGF-BB melanoma cell lines, were cultured in Dulbecco's Modified Eagle Media (DMEM; Sigma) containing 10% fetal bovine serum (FBS; 20% for EO771 cells), 100 units penicillin and 100 μg/mL streptomycin in a humidified incubator at 37°C. The medium used to culture the B16 and B16/PDGF-BB melanoma cell lines was supplemented with 150 μg/mL zeocine. PAE-PDGFRα cells were cultured in F12 media (Sigma), supplemented with 10% FBS and 2 mM L-glutamine. For serum starvation, all cells were cultured overnight in medium containing 0.1% FBS.

### PDGFRβ+ cell isolation

LLC and B16/BB tumors from all three treated experimental groups (vehicle, 1-NaPP1, imatinib) were excised and minced, following heart perfusion with PBS and PFA. The tissue was then digested for 20 minutes at 37°C with constant stirring with collagenase A (Roche), Hyaluronidase (Sigma), and DNase I (Invitrogen) dissolved in Hank's Balanced Salt Solution (Gibco/Invitrogen) supplemented with 0.1% BSA. PDGFRβ+ cells were isolated by overnight incubation with magnetic beads coated with biotinylated PDGFRβ antibodies (BD Biosciences). RNA extraction was then performed of the isolated population and the purity of the fraction was analyzed by quantitative PCR.

### Quantitative PCR

Total RNA was extracted from B16, EO771 and LLC cells with the RNeasy Mini kit (Qiagen). One μg (or 500 ng) of total RNA was reverse-transcribed using IScript cDNA synthesis kit (Bio-Rad) to create cDNA templates. Quantitative PCR was performed using KAPA SYBR Fast qPCR Kit (Kapa Biosystems) in triplicates by the CFX96 system (Bio-Rad) according to the manufacturer's instructions. Expression levels of *Pdgf-b* and *chondroitin sulfate proteoglycan* (*Cspg4*)*, X-linked inhibitor of apoptosis protein* (*Xiap*) and *vascular endothelial growth factor-a (Vegf-a)* were quantified by using *glyceraldehyde-3-phosphate dehydrogenase* (*Gapdh*) and *mitochondrial ribosomal protein L19* as housekeeping reference genes, respectively. The primer sequences for *Cspg4, Xiap* and* Vegf-a* are shown in **Table S1**, whereas the primers for *L19*, *Gapdh* and *Pdgf-b* have been reported previously 33,34.

### Immunostaining

Twelve μm cryosections were fixed with ice-cold acetone, methanol or 4% PFA. After blocking with serum-free protein block (Dako) or 5% donkey serum in PBS for 90 min at room temperature, the sections were incubated overnight at 4°C in a humidified dark chamber with primary antibodies (shown in **Table S2**) in PBS supplemented with 1% bovine serum albumin (BSA). Samples were then washed three times with PBS-1% BSA, incubated with appropriate Alexa conjugated fluorescent secondary antibodies (Life Technologies) for >1 h at room temperature, washed three times in PBS supplemented with 1% BSA, and finally mounted in Vectashield DAPI-containing mounting medium (Vector Laboratories).

### Image analysis

Imaging was performed using an Axio Imager M2 (Zeiss) with an AxioCam MRm digital camera and the ZEN 2012 software. Vascular parameters were measured using the AngioTool software, which can be used to determine morphological and spatial parameters, such as the overall size of the vascular network, the total and average vessel length, and vessel junctional density. Quantification of pericyte coverage, vessel perfusion and vessel apoptosis was performed using the open-source CellProfiler software version 2.2.0 [http://www.cellprofiler.org; [31]].

### Immunoblotting

Subconfluent cells were starved overnight and then stimulated for different time periods with 20 ng/mL PDGF-BB. In case of treatment with inhibitors, the cells were incubated for the indicated times with either vehicle (dimethyl sulphoxide; DMSO) or the inhibitors mentioned in **Table S3**, 1 h prior to stimulation with PDGF-BB. The stimulation was stopped by washing cells twice in ice-cold PBS. Cell lysis, SDS-PAGE and immunoblotting were performed as described elsewhere 35. The antibodies used for immunoblotting are described in **Table S2**.

Liquid nitrogen frozen tumors from mice treated with vehicle, imatinib or 1-NaPP1 were grinded with mortar and pestle, and tumor lysates were prepared in RIPA buffer (0.5% deoxycholate, 0.1% sodium dodecyl sulphate (SDS), 1% Triton X-100, 10% glycerol, 20 mM Tris, pH 7.4, 150 mM NaCl), supplemented with 100x Halt protease and phosphatase inhibitor cocktail (ThermoFisher), 1 mM Pefa Block, 1 mM sodium orthovanadate and 1 mM EDTA. Lysates were then centrifuged at 16000 x g for 30 minutes at 4^o^C, supernatants were collected and protein concentration was measured by using BCA protein assay (Pierce, Rockford, IL). Incubation with agarose bound WGA beads (Vector Laboratories) was performed overnight, followed by three washing steps with lysis buffer. Retained proteins were desorbed by boiling for 5 minutes in SDS sample buffer containing 10 mM dithiothreitol (DTT), and subjected to SDS-PAGE, followed by immunoblotting, as described elsewhere 35.

### Statistical analysis

Statistical analyses were carried out using GraphPad Prism version 7.0. The statistical significance of differences among mean values was determined by one-way ANOVA analysis and two-tailed t-test; *, p <0.05; **, p < 0.01; ***, p < 0.001. All the sample sizes were tested for normality and were appropriate for assumption of normal distribution and variance was similar between the groups. In one case, in which the sample size was tested negative for normality, Kruskal-Wallis and Mann-Whitney non-parametric tests were performed (Figure 2SI). *In vitro* analyses were repeated at least three times.

## Results

### The ASKA PDGFRβ kinase activity is inhibited by both 1-NaPP1 and imatinib

**Figure 1:**
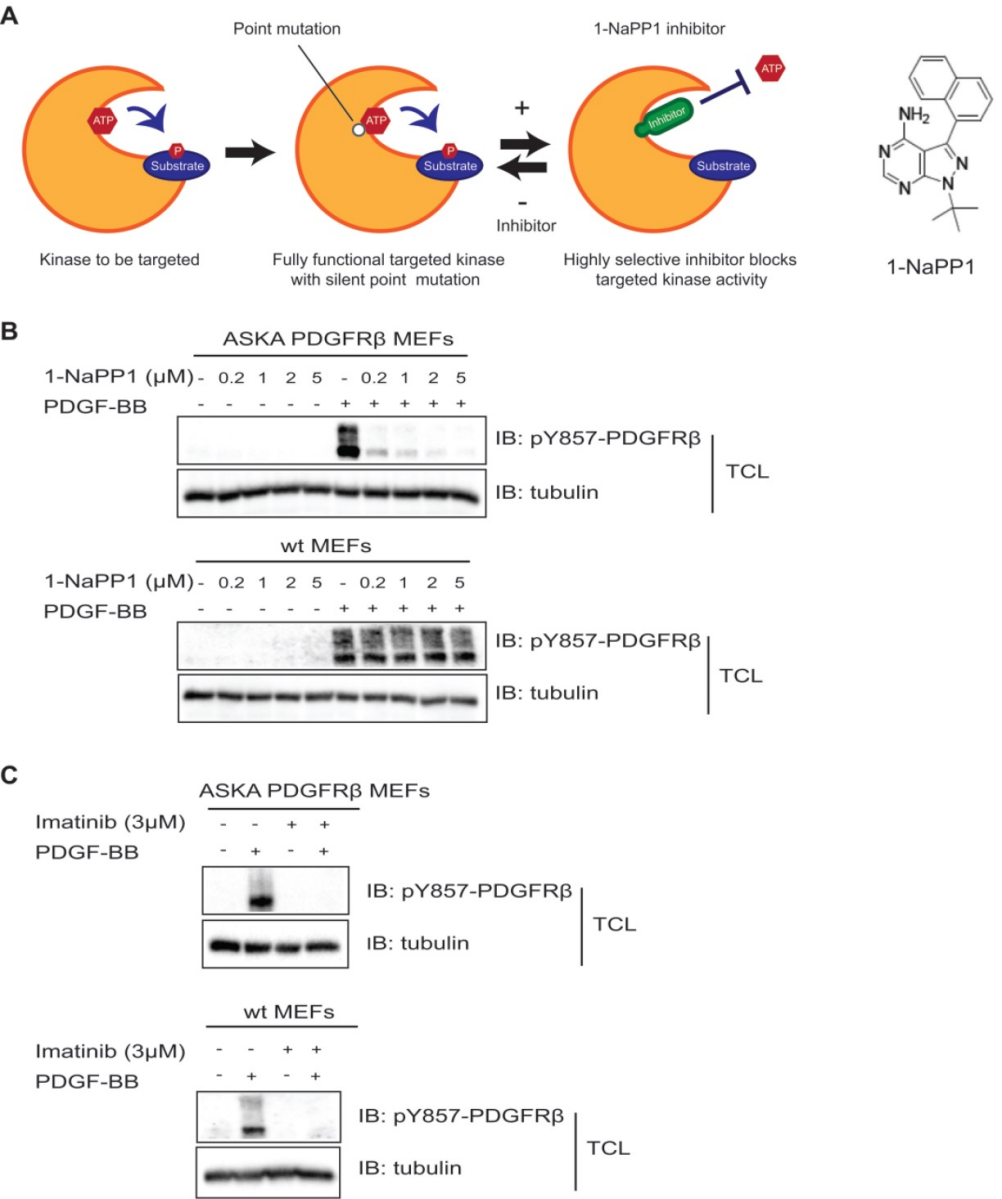
** Inhibition of PDGFRβ kinase activity by 1-NaPP1 and imatinib in wild-type and ASKA PDGFRβ MEFs.** (**A**) Schematic illustration of the PDGFRβ kinase switch mouse model carrying a silent point mutation from threonine to alanine in codon 680 of the ATP binding pocket of PDGFRβ. (**B**, **C**) ASKA and wild-type MEFs were serum-starved overnight and pre-treated with different concentrations of 1-NaPP1 (**B**) or 3 µM imatinib (**C**) for 1 h at 37°C, and then stimulated with 20 ng/mL PDGF-BB for 10 min. Total cell lysates were collected and PDGFRβ kinase activity was evaluated by immunoblotting (IB) using a pY857 PDGFRβ antibody. α-tubulin was used as a loading control. Panels B and C show representative immunoblots out of three independent experiments.

In the present study, we made use of a PDGFRβ kinase switch mouse model (Taconic Artemis). This model consists of a mouse line carrying a threonine to alanine point mutation at codon 680 in the ATP-binding pocket of PDGFRβ. Receptors harboring this mutation show specific inhibition of the PDGFRβ kinase upon administration of a specific compound, 1-NaPP1 (**Figure 1A**).

To confirm that the PDGFRβ kinase is functional in this mouse model and to investigate whether it could be inhibited by 1-NaPP1 and other PDGFRβ kinase inhibitors, we isolated MEFs from ASKA and wild-type littermate mice for *in vitro* analysis. PDGF-BB stimulation induced PDGFRβ autophosphorylation in both wild-type and ASKA mutant MEFs to the same extent. PDGF-BB-induced phosphorylation of PDGFRβ in wild-type MEFs was not affected by treatment with 1-NaPP1, while it was inhibited in ASKA mutant MEFs in a dose-dependent manner (**Figure 1B**). Furthermore, 1-NaPP1 was found not to inhibit PDGFRα in porcine aortic endothelial cells (**Figure S1A**). Imatinib inhibited the PDGF-BB-induced phosphorylation of PDGFRβ in both wild-type and ASKA MEFs (**Figure 1C**).

Since 1-NaPP1 selectively inhibited the kinase activity of ASKA PDGFRβ, the ASKA PDGFRβ mutant mouse model represents a useful tool to dissect the impact of exclusively targeting PDGFRβ kinase activity.

### Selective inhibition of PDGFRβ inhibits growth of LLC and B16/PDGF-BB tumors

Overexpression of PDGF-BB in tumor cells has been shown to generate increased pericyte coverage in solid tumors, resulting in better vessel perfusion and increased tumor growth 36. High tumor PDGF-BB expression has been shown to be associated with increased angiogenesis and to correlate with decreased patient survival 37. We compared tumor cells expressing high levels of PDGF-BB, such as Lewis Lung carcinoma cells (**Figure S1B** and **S1F**) and B16 melanoma cells stably transfected with PDGF-BB (**Figure S1C**) 38, with tumors with low PDGF-BB content (B16 melanoma and EO771; **Figure S1D-F**). Previous studies have reported that LLC and B16 cell lines do not express PDGFRβ 39,40; by immunoblotting of lysates of cultured cells we found low (B16, B16/PDGF-BB, EO771) or no (LLC) expression of PDGFRβ on these cells (**Figure S1G**). Additionally, all tumor models are syngeneic to C57BL/6 background, the same genetic background as the ASKA mutant mice; tumor cells were grown subcutaneously in the ASKA PDGFRβ mice, apart from EO771 that was orthotopically injected into the 4^th^ mammary fat pad.

**Figure 2:**
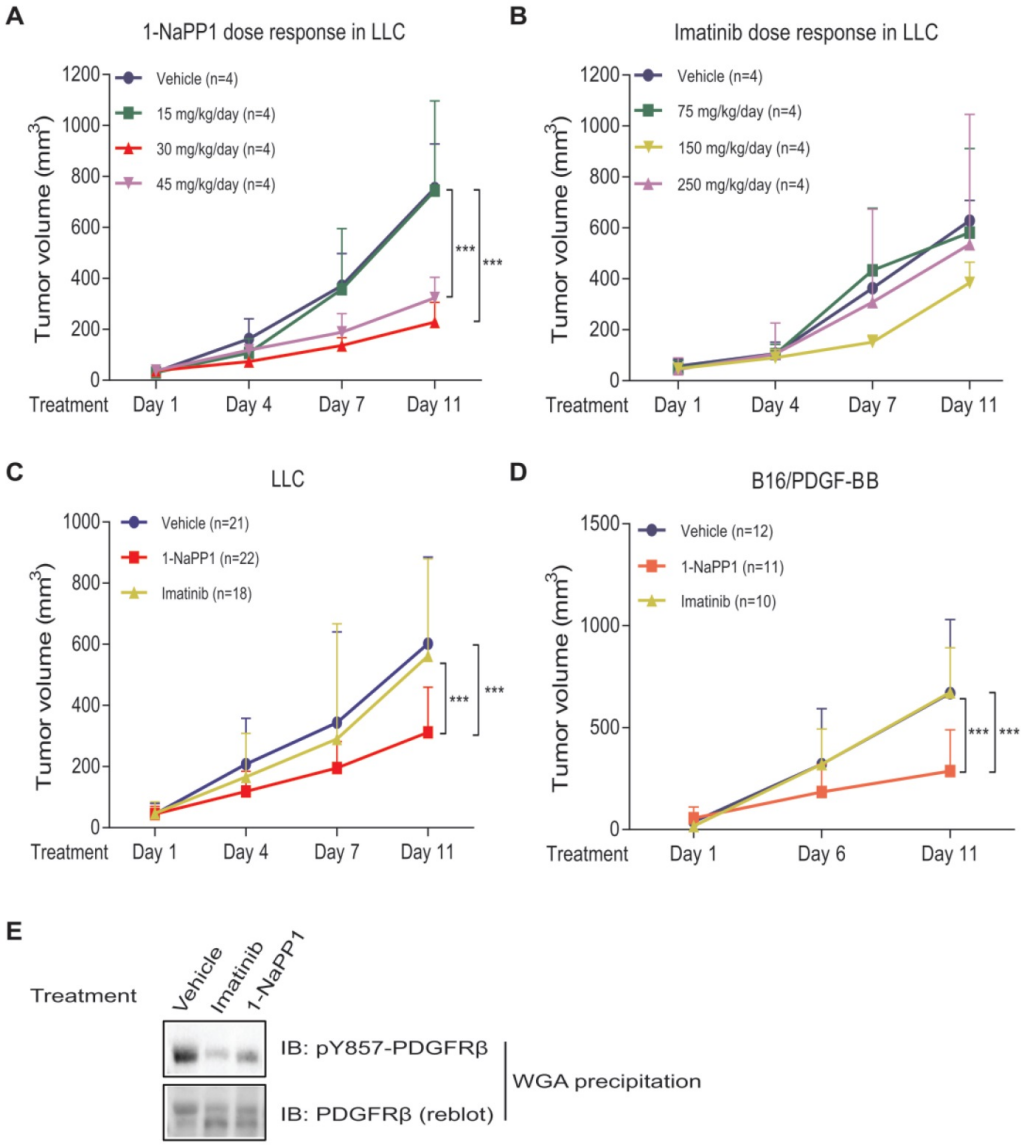
** Selective inhibition of PDGFRβ inhibits growth of LLC and B16/PDGF-BB tumors with paracrine PDGF-BB signaling.** (**A**, **B**) Dose-response analysis of treatment with 1-NaPP1 (15, 30 and 45 mg/kg/day) (**A**) or imatinib (75, 150 and 250 mg/kg/day) (**B**) of mice with Lewis lung carcinoma cells grown subcutaneously. (**C**, **D**) Effect of 1-NaPP1 (30 mg/kg/day) and imatinib (150 mg/kg/day) on growth of LLC (**C**) and B16/PDGF-BB tumors (**D**) in ASKA mice for 10 days (n= as stated in the figure). *** p<0.001. (**E**) LLC tumor lysates from mice treated with vehicle, 1-NaPP1 (30 mg/kg) or imatinib (150 mg/kg) were prepared and incubated overnight with WGA-beads. Retained proteins were analyzed by immunoblotting (IB) using a pY857 PDGFRβ antibody. Representative immunoblots out of two independent experiments are shown.

To identify the 1-NaPP1 dose that efficiently inhibits PDGFRβ signaling, we tested three different doses, one that should keep detectable levels of 1-NaPP1 in the mouse circulation for 24 h, i.e. 30 mg/kg of body weight (data not shown, Taconic Artemis communication), as well as a lower (15 mg/kg) and a higher (45 mg/kg) dose. After 10 days of treatment, both doses of 30 and 45 mg/kg had caused significant tumor growth impairment (p<0.001) with the most efficient inhibition of tumor progression by 30 mg/kg, while the lowest dose provided no therapeutic benefit (**Figure 2A**).

In most animal studies with imatinib, a standard dosage of 150 mg/kg body weight per day is used. In order to determine the optimal dose for ASKA PDGFRβ mutant mice with LLC tumors, we investigated in addition to the 150 mg/kg, also 75 mg/kg and 250 mg/kg per day. This dose-response analysis revealed that 150 mg/kg/day gave a small but not significant reduction in LLC tumor growth, whereas doses of 75 mg/kg and 250 mg/kg had no effect on tumor growth (**Figure 2B**). Based on these results we selected daily doses of 30 mg/kg for 1-NaPP1 and 150 mg/kg for imatinib for all subsequent experiments.

We next investigated the effect of specifically inhibiting PDGFRβ kinase activity using 1-NaPP1, in comparison with the less selective kinase inhibitor imatinib. After 10 days of daily treatment, 1-NaPP1 suppressed growth of LLC tumors in the ASKA PDGFRβ mutant mouse model (**Figure 2C**) compared to vehicle-treated tumors (p<0.001), while imatinib did not exhibit any appreciable therapeutic benefit. Strikingly, the growth tumors expressing low amounts of PDGF-BB, such as B16 melanoma and EO771 breast cancer tumors, were not affected by treatment with 1-NaPP1 or imatinib (**Figure S2A-B**). Given these observations, we further investigated the possibility that targeting PDGFRβ kinase activity only renders therapeutic benefit in the presence of enhanced PDGF-BB/PDGFRβ signaling. Similarly, to the LLC tumors, 10 daily treatments of the PDGF-BB overexpressing tumor model B16/PDGF-BB with 1-NaPP1 unveiled a decreased tumor growth rate (p<0.001) in the ASKA PDGFRβ mutant mouse model, whereas imatinib gave no effect (**Figure 2D**). To confirm that the kinase activity of PDGFRβ was inhibited *in vivo*, we analyzed LLC tumor lysates from mice treated with vehicle, 1-NaPP1 or imatinib, by immunoblotting using an antibody against pTyr857; the PDGF-BB-induced phosphorylation of PDGFRβ was suppressed in mice treated with either 1-NaPP1 or imatinib (**Figure 2E**).

Thus, selective inhibition of host PDGFRβ in ASKA mice by 1-NaPP1 significantly inhibited tumor growth in LLC and B16/PDGF-BB tumor models that express high levels of PDGF-BB. Surprisingly, imatinib, which has a broader inhibitory profile, did not affect tumor growth in either of the tumor models studied.

### Selective inhibition of PDGFRβ impairs tumor vascularization in LLC and B16/PDGF-BB tumors

**Figure 3:**
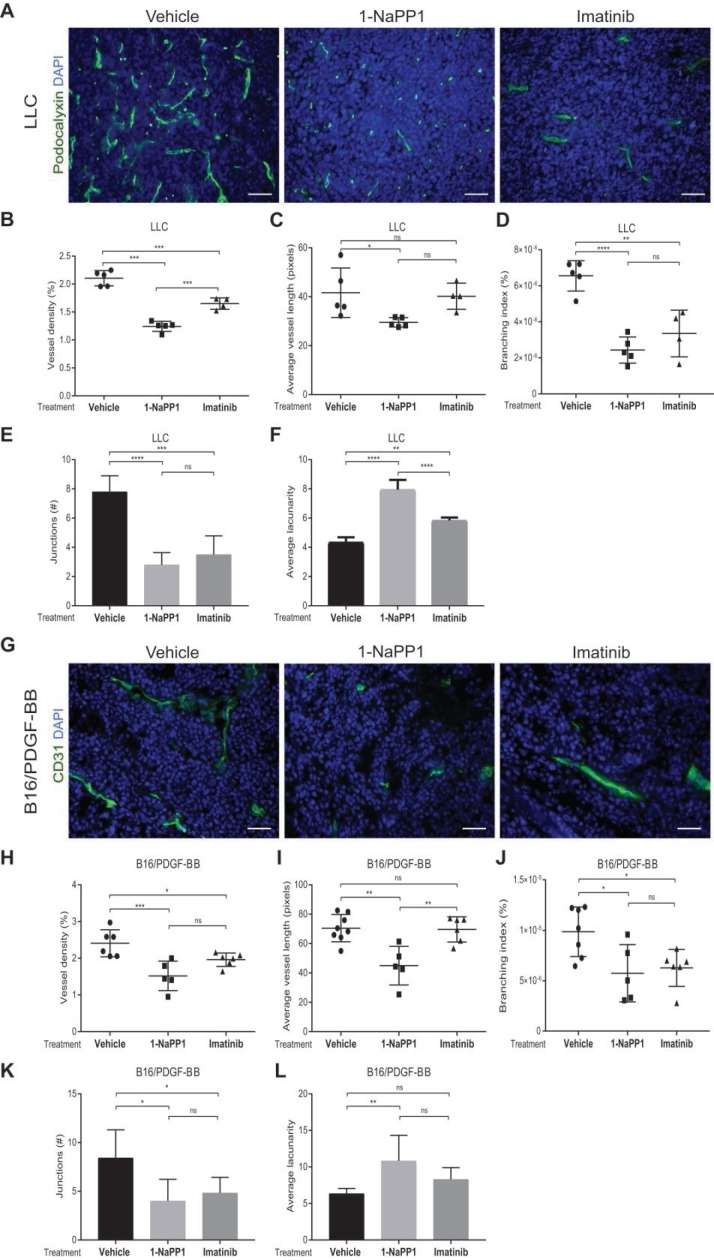
** Selective inhibition of PDGFRβ impairs tumor vascularization in LLC and B16/PDGF-BB tumors.** LLC (**A-F**) and B16/PDGF-BB (**G-L**) tumors were grown in ASKA PDGFRβ mutant mice. After treatment with 1-NaPP1 or imatinib for 10 consecutive days, sections from tumors were immunostained for podocalyxin (LLC tumors; **A**) and CD31 (B16/PDGF-BB; **G**); podocalyxin/CD31, green; DAPI, blue; >20 field 200x magnification images were scored for each mouse (n=5 or more animals). Scale bar, 50 µM. Vascular parameters were analyzed using the Angiotool software. Vessel density expressed as a percentage of the tumor area (**B**, **H**), average vessel length (**C**, **I**), branching (junction density; **D**, **J**), number of junctions (**E**, **K**) and average lacunarity (**F**, **L**) of LLC (**B-F**) and B16/PDGF-BB tumors (**H-L**), are shown. *p<0.05, **p<0.01, ***p<0.001 and ****p<0.0001. Each data point corresponds to one individual mouse (n=5 or more animals).

Given the well-documented importance of PDGF-BB and PDGFRβ signaling for pericyte recruitment to the vasculature, we analyzed the effects of impairing PDGFRβ activity on the tumor vasculature. Vasculature was visualized by CD31 or podocalyxin immunostainings and vascular parameters, including vessel density, average vessel length and vessel branching, were measured using Angiotool, a software validated for studies on angiogenesis and vascular development 41. Immunostaining of LLC tumors for the endothelial marker podocalyxin showed that vessel density, vessel branching and number of vessel junctions, were significantly decreased (**Figure 3A-B and 3D-E**) by either 1-NaPP1 or imatinib. In contrast, the vascular lacunarity, i.e. the irregularity and the size of gaps between blood vessels, was significantly higher after treatment with 1-NaPP1 than imatinib (**Figure 3F**). Moreover, specific targeting with 1-NaPP1 elicited a much stronger effect on the vascular tree compared to imatinib, as a significant decrease in vessel length was observed compared to both vehicle- and imatinib- treated tumor vessels (**Figure 3C**). Analysis of several tumors revealed a positive correlation between tumor volume and vessel density (**Figure S3A**). Furthermore, similar analysis of the B16/PDGF-BB tumor vasculature with the widely used vascular markers CD31 (**Figure 3G**) and podocalyxin (data not shown) revealed an inhibitory effect of 1-NaPP1 on vessel density, length, branching and number of junctions as well as a significant increase in vascular lacunarity, thus corroborating the strong impact on the vasculature upon PDGFRβ signaling inhibition (**Figure 3H-L**). In contrast, the tumor vasculature (**Figure S2C-H**) was neither significantly affected by 1-NaPP1 nor by imatinib in B16 tumors having low expression of PDGF-BB, although a trend towards decreased vessel density was observed (**Figure S2C-D)**.

In conclusion, inhibition of PDGFRβ signaling strongly affects the vascularization of LLC and B16/PDGF-BB tumors, most likely through interfering with pericyte function and concomitant negative effects on the endothelium.

### Selective inhibition of PDGFRβ impairs vascular function and increases vessel apoptosis in tumors with high PDGF-BB expression

**Figure 4:**
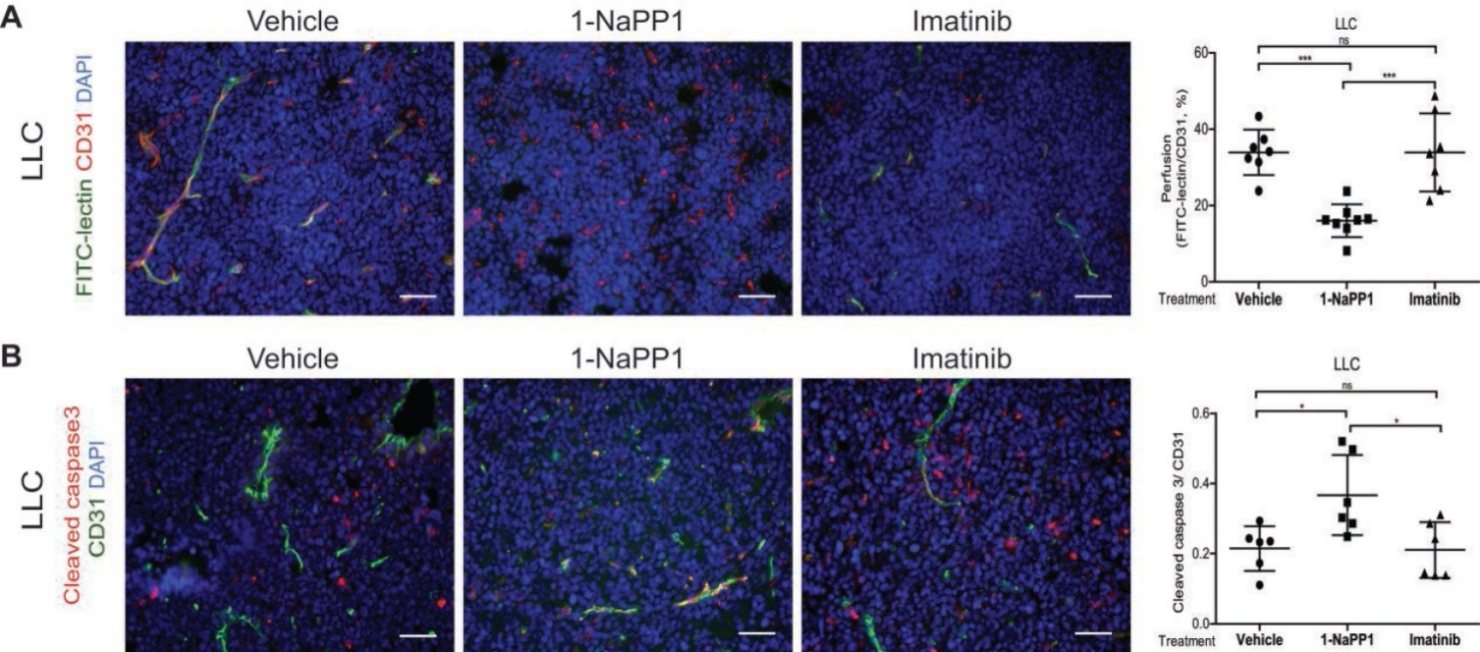
** Selective inhibition of PDGFRβ impairs vascular function and increases vessel apoptosis in tumors with high PDGF-BB expression.** (**A**) Tumor blood vessel perfusion was assessed by injection of FITC-lectin in LLC tumor-bearing ASKA mice treated with vehicle, 1-NaPP1 or imatinib daily for 10 consecutive days. Tumor sections were then stained for CD31 and vessel perfusion was examined in more than 20 field 200x magnification images for each mouse (n=6 or more animals); FITC-lectin, green; CD31, red; DAPI, blue. Scale bar, 50 µM. Quantification of vessel perfusion is shown where each data point corresponds to one individual mouse. ***p<0.001. (**B**) Endothelial cell apoptosis was analyzed by co-immunostaining of cleaved caspase 3 and CD31; cleaved caspase 3, red; CD31, green; DAPI, blue. Quantification of the vessels positive for the apoptosis marker is depicted. *p<0.05; each data point corresponds to one individual mouse (n=6 or more animals).

Since selective targeting of PDGFRβ by 1-NaPP1 strongly affected tumor growth and vasculature leading to a significant decrease in vessel size in both LLC and B16/PDGF-BB tumors, we studied the effect of 1-NaPP1 treatment on tumor vasculature perfusion. By injection of FITC-conjugated lectin upon treatment with 1-NaPP1, a significantly impaired vascular perfusion was seen, compared with both vehicle and imatinib-treated tumors (**Figure 4A**). As vessel size and perfusion were strongly affected in 1-NaPP1-treated LLC tumors, we further examined the possibility that endothelial cells undergo apoptosis in the presence of 1-NaPP1. Immunostaining for the vessel marker CD31 and the apoptosis marker cleaved caspase 3 revealed a significant increase in apoptotic endothelial cells after 1-NaPP1 treatment, but not after treatment with vehicle or imatinib (**Figure 4B)**. This observation suggests that selective PDGFRβ inhibition by 1-NaPP1 prevents tumor growth by inducing endothelial cell apoptosis. Analysis of several tumors showed positive and negative correlations between vessel perfusion and tumor volume (**Figure S3B**) and endothelial cell apoptosis (**Figure S3C**), respectively.

### Selective inhibition of PDGFRβ differentially affects tumor pericyte populations depending on PDGF-BB levels

**Figure 5:**
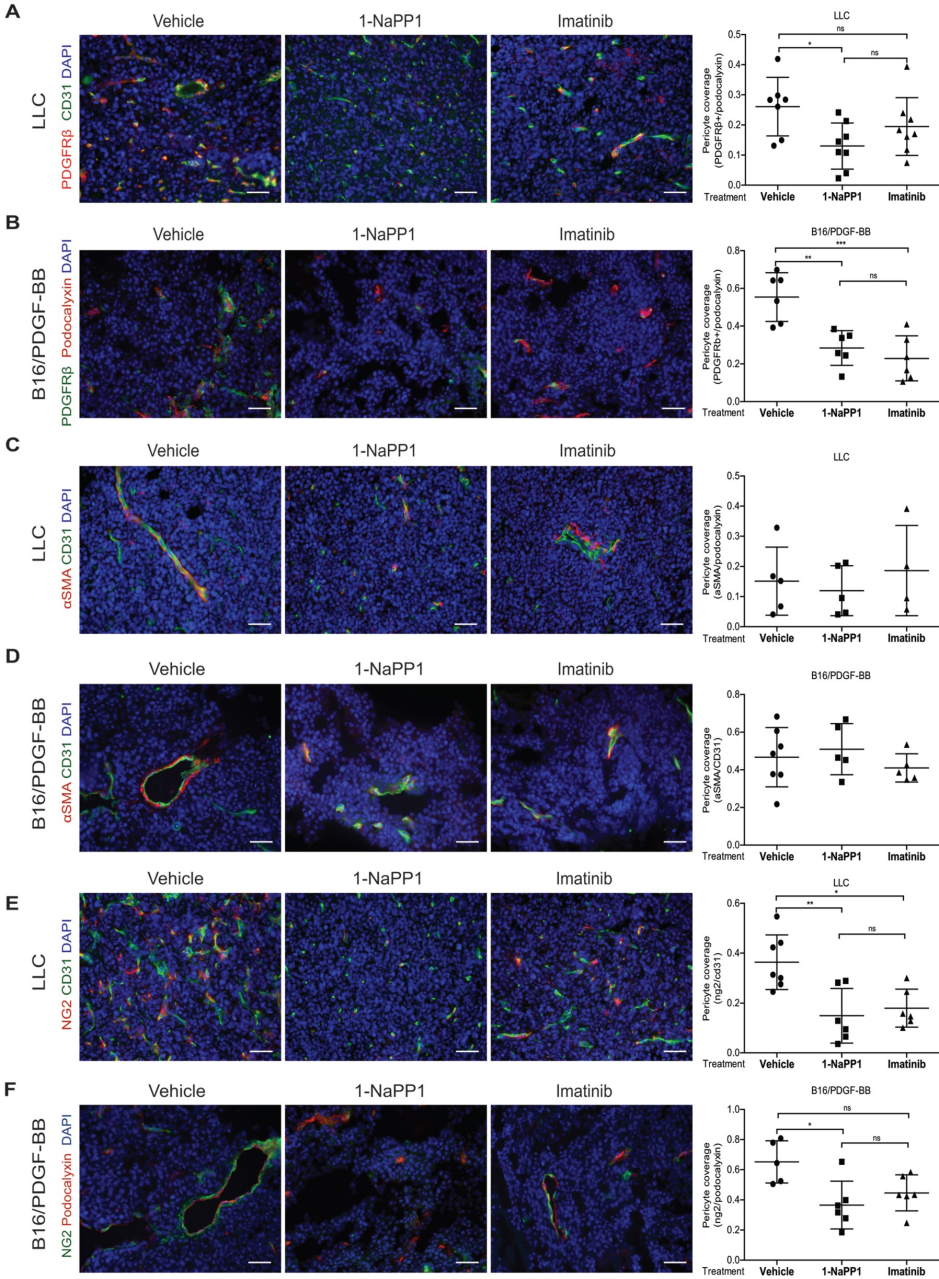
** Selective inhibition of PDGFRβ differentially affects tumor pericyte populations in LLC and B16/PDGF-BB tumors.** LLC (**A**, **C**, **E**) and B16/PDGF-BB (**B**, **D**, **F**) tumors were grown in ASKA PDGFRβ mutant mice after treatment with vehicle, 1-NaPP1 or imatinib for 10 consecutive days; sections from tumors were co-immunostained for CD31/podocalyxin and PDGFRβ. PDGFRβ+ pericyte coverage was quantified in LLC (**A**; CD31, green; PDGFRβ, red) and B16/PDGF-BB (**B**; podocalyxin, red; PDGFRβ, green). CD31 and α-SMA were co-immunostained and α-SMA+ pericyte coverage quantified in LLC (**C**) and B16/PDGF-BB (**D**) tumors (CD31, green; α-SMA, red). Podocalyxin or CD31 and NG2 were co-immunostained and NG2+ pericyte coverage quantified in LLC (**E**) and B16/PDGF-BB (**F**) tumors (LLC: CD31, green; NG2, red; B16/PDGF-BB: podocalyxin, red; NG2, green). >20 field 200x magnification images were scored for each mouse (n=5 or more animals). Scale bar, 50 µm. *p<0.05, **p<0.01 and ***p<0.001.

We next evaluated the impact of inhibiting PDGFRβ kinase activity on the PDGFRβ+ pericyte population. Targeting of PDGFRβ kinase activity led to a significant decrease of PDGFRβ+ pericyte coverage of tumor vessels in LLC and B16/PDGF-BB tumors treated with either 1-NaPP1 or imatinib (**Figure 5A and B**). However, neither 1-NaPP1 nor imatinib influenced the α-SMA+ pericytes of LLC (**Figure 5C**) or B16/PDGF-BB tumors (**Figure 5D**), probably because this marker is most predominantly expressed in high caliber well established vessels, and therefore less sensitive to the inhibitors. In contrast, a significant reduction in NG2+ pericyte coverage of tumor vessels was seen in both LLC and B16/PDGF-BB tumors treated with 1-NaPP1 (**Figure 5E-F**). Treatment with imatinib also suppressed NG2+ pericyte coverage, but only in LLC tumors.

B16 tumors, which comprise low PDGF-BB paracrine signaling, showed a trend towards decreased PDGFRβ+ pericyte coverage of tumor vessels after treatment with 1-NaPP1 as well as imatinib, whereas no change was observed on NG2+ pericyte coverage (**Figure S2I-J**).

To summarize, selective and unselective impairment of PDGFRβ kinase function rendered similar effects on the tumor pericyte populations in the ASKA PDGFRβ mutant mice, although in most cases stronger effects were observed upon selective PDGFRβ signaling inhibition. While α-SMA+ pericyte coverage seemed unaffected by either treatment, a reduction of PDGFRβ+ pericytes was shown in the presence of either of the two PDGFRβ kinase inhibitors. Interestingly, upon PDGFRβ kinase inhibition the NG2+ pericyte coverage was substantially reduced, suggesting a role of PDGFRβ signaling in the regulation of NG2 expression.

### PDGF-BB induces expression of NG2 in pericyte precursor cells

**Figure 6:**
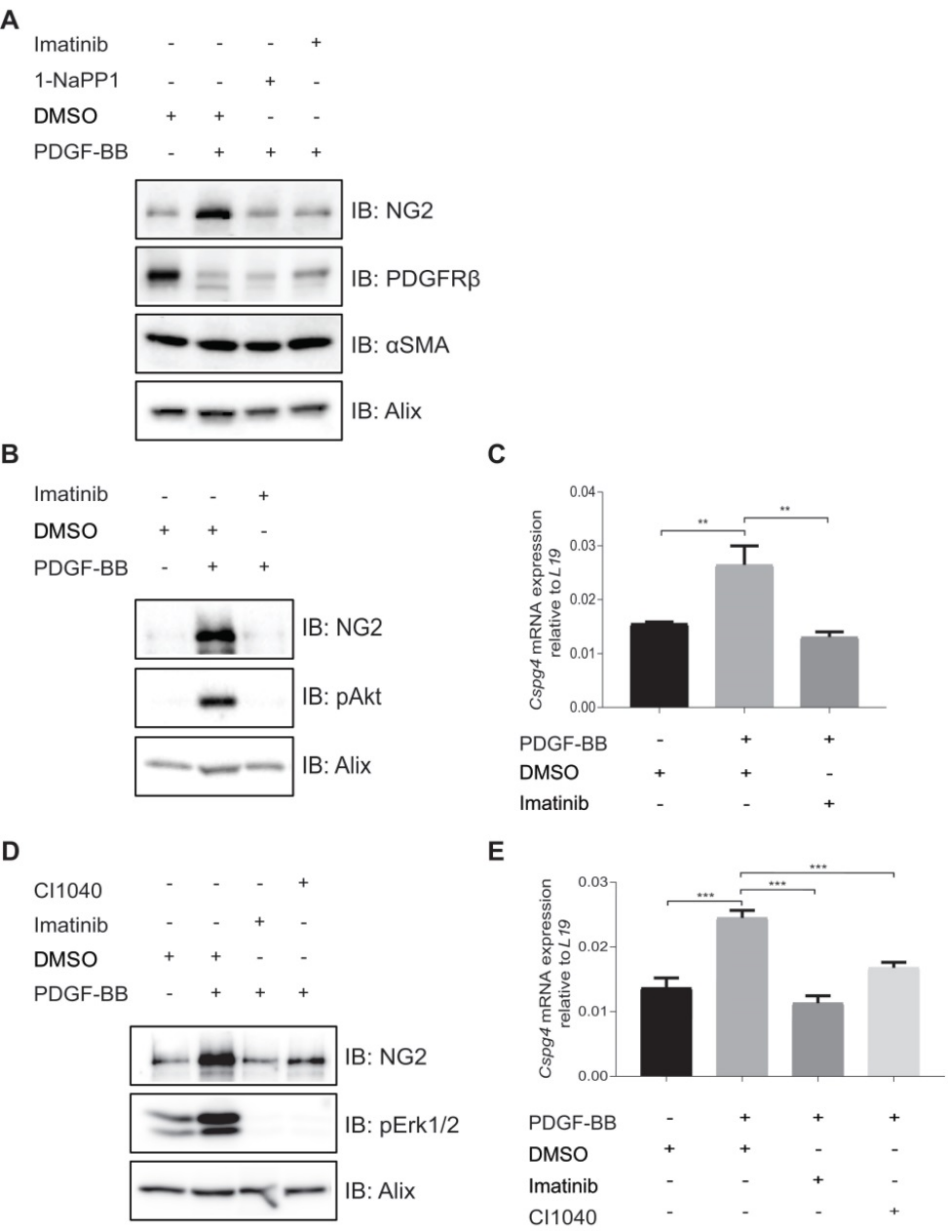
***In vitro* inhibition of PDGFRβ affects expression of pericyte markers in ASKA MEFs and 10T1/2 cells.** (**A**) Immunoblotting of pericyte markers in ASKA MEFs, serum-starved and treated with either DMSO, 1-NaPP1 (1 µM) or imatinib (3 µM) in the presence or absence of 20 ng/mL PDGF-BB for 24 h. Total cell lysates were collected and subjected to SDS-PAGE, followed by immunoblotting (IB) for different pericyte markers (NG2, PDGFRβ and α-SMA). IB for Alix was used as a loading control. Representative immunoblots out of three independent experiments, are shown. (**B**) Immunoblotting for NG2 pericyte marker in 10T1/2 cells, serum-starved and treated with DMSO or imatinib (3 µM) in the presence or absence of 20 ng/ml PDGF-BB for 24 h. Total cell lysates were collected and subjected to SDS-PAGE and the expression of NG2 and phosphorylated pAkt were evaluated by IB. IB for phosphorylated Akt was used to verify receptor stimulation and IB for Alix to verify equal protein loading. Experiment was performed in triplicates and representative immunoblots are presented. (**C**) To measure *Cspg4* (NG2) mRNA expression in 10T1/2 cells, serum-starved and treated with DMSO or imatinib (3 µM) in the presence or absence of 20 ng/ml PDGF-BB for 24 h, we performed quantitative real-time PCR. Error bars indicate standard deviation from triplicate samples. All mRNA expression is relative to *L19* ribosomal gene expression. Three independent experiments were performed and statistical analysis was performed by using *student t-test*. **p<0.01. (**D**) PDGF-BB induces NG2 expression in a Mek1/2-dependent manner. 10T1/2 cells were serum-starved and treated for 24 h with inhibitors targeting PDGFRβ kinase activity (imatinib, 3 µM) or Mek1/2 (CI-1040, 3 µM) in the presence or absence of 20 ng/mL PDGF-BB. Total cell lysates were collected and the expression of NG2 and phosphorylated pErk1/2 were evaluated by immunoblotting. IB for Alix was used as a loading control and IB for pErk1/2 as a control for the effect of CI1040. Representative immunoblots out of three independent experiments, are shown. (**E**) To measure *Cspg4* mRNA expression, we performed quantitative real-time PCR. Error bars indicate standard deviation from triplicate samples. All mRNA expression is relative to *L19* ribosomal gene expression. Three independent experiments were performed and statistical analysis was performed by using *student t-test*. ***p<0.001.

To further investigate the effects of 1-NaPP1 and imatinib on the expression of NG2, we employed PDGFRβ mutant ASKA MEFs. PDGF-BB stimulation of these cells promoted a substantial increase in NG2 protein levels, which was strongly blunted by 1-NaPP1 treatment, suggesting that PDGF-BB promotes NG2+ pericyte differentiation (**Figure 6A**).

We further attempted to validate the effect of PDGF-BB on NG2 expression in MOVAS vascular smooth muscle cells, HBVP brain pericyte primary cells and the 10T1/2 pericyte precursor cell line. PDGF-BB only induced NG2 expression in the mesenchymal pericyte precursor cell line 10T1/2 (**Figure 6B-C**), suggesting that only mesenchymal precursor cells still retain the capacity to induce NG2 expression upon PDGF-BB stimulation. Using a panel of selective inhibitors that target different signaling pathways known to function downstream of PDGFRβ, we found that NG2 expression in response to PDGF-BB stimulation was inhibited by the Mek1/2 inhibitor CI-1040 (**Figure 6D-E**), suggesting a role for Mek/Erk1/2 pathway in the regulation of PDGF-BB-induced NG2 expression.

## Discussion

The aim of the present study was to dissect the effects of specific targeting of the PDGFRβ kinase activity on tumor growth and vascularization. We studied cancer cell lines with no or low PDGFRβ expression, and used the ASKA mouse model, which provided an opportunity to elucidate the specific role(s) of PDGFRβ signaling in the host during tumor growth and explore the value of highly selective PDGFRβ kinase inhibitors as anti-cancer drugs.

We found that specific inhibition of PDGFRβ activity strongly reduced tumor growth and angiogenesis in tumors with high PDGF-BB expression, while the less specific kinase inhibitor imatinib rendered negligible suppression of tumor growth even though it affected the vascularization of tumors. Indeed, it has previously been shown that imatinib monotherapy does not inhibit tumor growth in different tumor models, such as the B16/PDGF-BB melanoma 38, the human gastric carcinoma orthotopic nude mouse xenograft 42, the pancreatic neuroendocrine tumor model 43 and the human MA-11 breast carcinoma 44. Imatinib is used as first line therapy in CML and GIST, based on its ability to target Bcr-Abl, c-Kit and PDGFRβ expressed by the tumor cells. It has also been reported to be efficacious as monotherapy in a cervical mouse model of cancer by targeting PDGFR signaling and infiltration of cancer-associated fibroblasts 22.

We found that tumors with high expression of PDGF-BB, *i.e*. LLC and B16/PDGF-BB, and hence elevated PDGFRβ kinase activity, did respond to 1-NaPP1 treatment in the ASKA mutant mice, whereas low PDGF-BB expressing tumors, such as B16 and EO771 did not. A previous report on tumor response and PDGF-BB dose-dependency suggested that anti-PDGF drugs had an inhibitory effect on tumors with high PDGF-BB content 45. One possible explanation for the dose-dependency is that tumors become addicted to high PDGF-BB levels and therefore susceptible to PDGFRβ inhibition, similar to the well-established oncogene addiction 46. The levels of tumor PDGF-BB may thus serve as a biomarker for selection of cancer patients for anti-PDGF therapy.

High PDGF-BB expression correlated with poor progression-free survival in a phase II clinical trial on patients with non-small cell lung cancer treated with imatinib 37, consistent with our findings that imatinib does not provide therapeutic benefit in the high PDGF-BB expressing lung cancer model LLC. However, our finding that selective targeting of PDGFRβ in cancer models with high PDGF-BB expression did have a therapeutic effect, suggests that specific PDGFRβ kinase inhibitors may have a value in cancer therapy. The lack of therapeutic benefit of imatinib is counter intuitive, since it inhibits the activity of PDGFRβ as well as other kinases 47, hence one may have expected stronger anti-tumor effect by imatinib treatment. The exact reason for the limited effect by imatinib remains to be explained, but it is possible that imatinib simultaneously inhibits both tumor promoting and inhibiting pathways induced by different kinases. The observation that 1-NaPP1, but not imatinib, induced apoptosis is compatible with the possibility that imatinib, but not 1-NaPP1, inhibits pro-apoptotic or enhances anti-apoptotic pathways. A potential mechanism for the differential effects on tumor angiogenesis and growth may be related to the observation that while both 1-NaPP1 and imatinib reduced the *Vegf-a* expression in cell lines and tumor tissue, only imatinib treatment upregulated the anti-apoptotic gene *Xiap* that could possibly protect from apoptosis (**Figure S4**). This finding is in concurrence with studies showing that expression of XIAP is associated with imatinib resistance in chronic myeloid leukemia 48,49.

Not much is known about the regulation of expression of different pericyte markers. Here we showed that PDGF-BB induced the expression of the pericyte marker NG2 in ASKA MEFs. Tumor pericyte markers are not mutually exclusive, and several different markers can be concomitantly expressed in the same pericyte. Interestingly, it has been shown that PDGFRβ-positive progenitor cells from the bone marrow are able to differentiate into α-SMA- and NG2-positive pericytes during tumor angiogenesis 50. It is plausible that 1-NaPP1 directly inhibits bone marrow PDGFRβ-positive progenitor cells, thereby preventing their differentiation into NG2-positive pericytes and PDGF-BB-induced pericyte recruitment to the tumor. Importantly, the induction of NG2 by PDGF-BB, or its suppression by PDGFRβ kinase inhibition, was only found in the ASKA MEFs and 10T1/2 cells, but not in differentiated perivascular cells such as human brain vascular pericytes (HBVP) or the vascular smooth muscle cells (MOVAS) (data not shown). One reason could be that the ASKA MEFs are still in a primary state maintaining mesenchymal precursor cell properties, while the other more differentiated cell lines are no longer prone to NG2 regulation *in vitro*.

A previous study using the pancreatic neuroendocrine tumor model Rip1Tag2 identified 3 populations of pericytes in pancreatic tumors based on expression of PDGFRβ and NG2: PDGFRβ+ only, NG2+ only, and cells expressing both PDGFRβ+ and NG2+ 50. Although PDGFRβ and NG2 expression does not always overlap, our results suggest that there is a strong link between PDGFRβ signaling and NG2 expression. These observations further suggest that PDGFRβ signaling is involved in the regulation and maintenance of at least some NG2-positive pericyte populations. However, the α-SMA-positive pericytes neither responded to selective nor to unselective PDGFRβ inhibition, even if bone marrow PDGFRβ+ progenitor cells have been shown to also differentiate into α-SMA-positive pericytes 50.

The pericytes sustain important support functions for the endothelial cells by providing both physical protection and scaffolding to the vasculature, but also through intimate cellular crosstalk with the endothelial cells through paracrine signaling. Since we could not observe a direct effect of 1-NaPP1 or imatinib on the expression of PDGFRs or VEGFR2 in endothelial cells (data not shown), it is likely that the observed effect on the vasculature is due to interference with the crosstalk between PDGFRβ expressing pericytes, or other PDGFRβ expressing cells, and endothelial cells. In fact, pericytes are known to provide important survival cues to the endothelium 51. This notion is supported by our finding that inhibition of PDGFRβ kinase activity with 1-NaPP1 led to a significant decrease in pericyte coverage, and to a significant increase in endothelial cell apoptosis. This observation was further supported by the strong inhibitory effect of 1-NaPP1 on vessel density, size and perfusion, suggesting that specific PDGFRβ inhibition impairs tumor growth by inducing endothelial cell apoptosis, and thus decreasing blood vessel function. Although imatinib-treated tumors also demonstrated a significant decrease in vessel density, no change was observed either on vessel size, perfusion or endothelial cell apoptosis. In this context, it is of interest to note that imatinib has been shown to promote vessel normalization, modulate the extracellular matrix composition, increase vessel perfusion, and increase the delivery of chemotherapeutic agents 52-55.

Our data support the notion that selective targeting of host PDGFRβ gives a treatment benefit in preclinical tumor models with strong paracrine PDGF-BB stimulation. Therefore, it is possible that treatment with specific PDGFRβ kinase inhibitors preferentially is effective for cancers with high PDGF-BB expression. Thus, for use of selective PDGFRβ future kinase inhibitors for treatment of epithelial tumors, stratification of patients based on tumor PDGF-BB expression may be necessary.
