# Specific targeting of PDGFRβ in the stroma inhibits growth and angiogenesis in tumors with high PDGF-BB expression: Erratum

**DOI:** 10.7150/thno.48039

**Published:** 2020-06-11

**Authors:** Maria Tsioumpekou, Sara I. Cunha, Haisha Ma, Aive Åhgren, Jessica Cedervall, Anna-Karin Olsson, Carl-Henrik Heldin, Johan Lennartsson

**Affiliations:** 1 Department of Medical Biochemistry and Microbiology, Uppsala University, Sweden.; 2 Department of Pharmaceutical Biosciences, Uppsala University, Sweden.; 3 Ludwig Institute for Cancer Research, Uppsala Branch, Uppsala University, Sweden.; 4 Department of Immunology, Genetics and Pathology, Uppsala University, Sweden.; 5 Department of Neuroscience, Uppsala University, Sweden.

We noticed an error in figure 5 in our published manuscript 1. In figure 5 one of the axis in the quantification has been labelled with the wrong marker. Below is a correct version of figure 5.

## Figures and Tables

**Figure 5:**
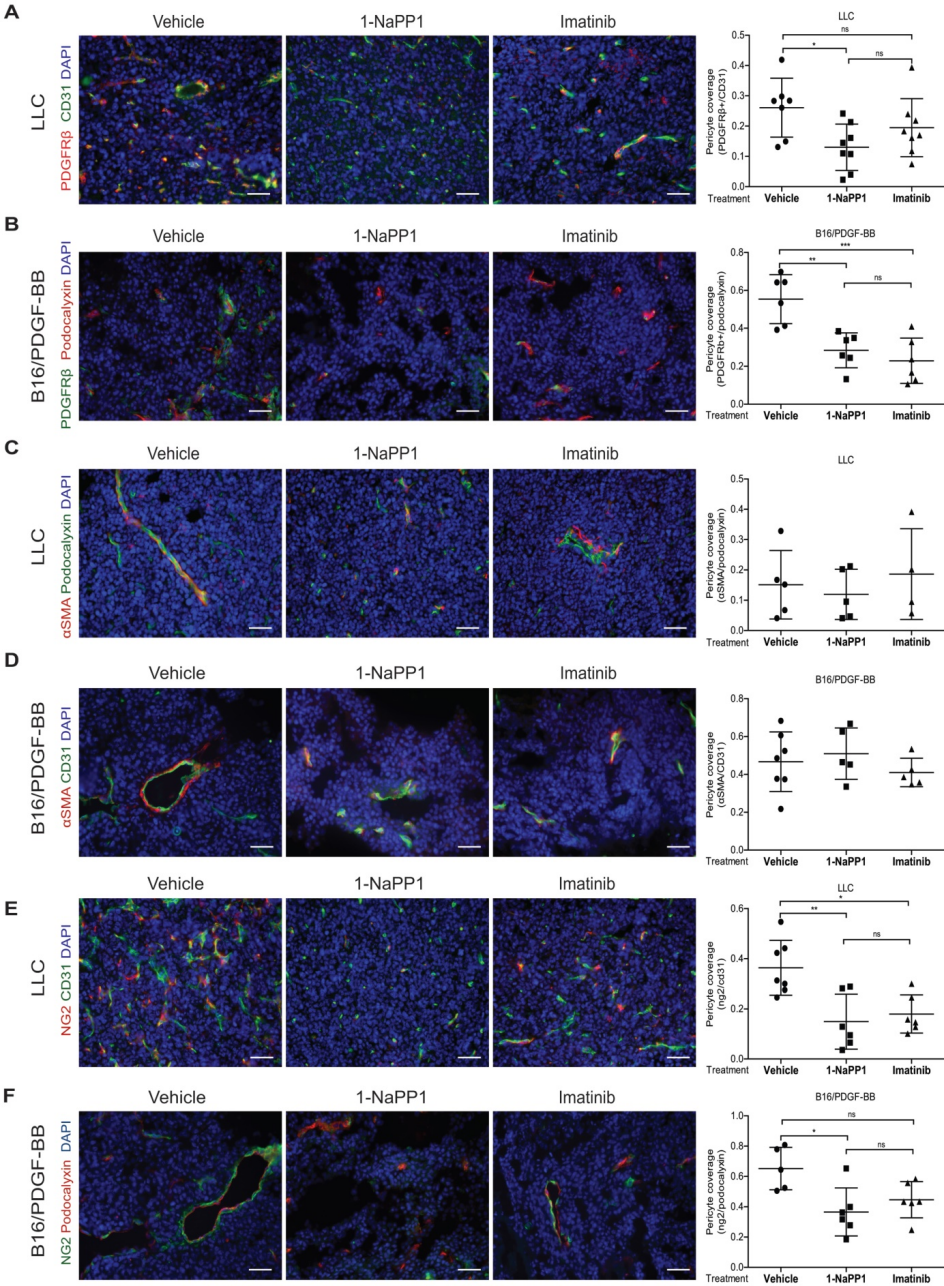
** Selective inhibition of PDGFRβ differentially affects tumor pericyte populations in LLC and B16/PDGF-BB tumors.** LLC (**A**, **C**, **E**) and B16/PDGF-BB (**B**, **D**, **F**) tumors were grown in ASKA PDGFRβ mutant mice after treatment with vehicle, 1-NaPP1 or imatinib for 10 consecutive days; sections from tumors were co-immunostained for CD31/podocalyxin and PDGFRβ. PDGFRβ+ pericyte coverage was quantified in LLC (**A**; CD31, green; PDGFRβ, red) and B16/PDGF-BB (**B**; podocalyxin, red; PDGFRβ, green). CD31 and α-SMA were co-immunostained and α-SMA+ pericyte coverage quantified in LLC (**C**) and B16/PDGF-BB (**D**) tumors (CD31, green; α-SMA, red). Podocalyxin or CD31 and NG2 were co-immunostained and NG2+ pericyte coverage quantified in LLC (**E**) and B16/PDGF-BB (**F**) tumors (LLC: CD31, green; NG2, red; B16/PDGF-BB: podocalyxin, red; NG2, green). >20 field 200x magnification images were scored for each mouse (n=5 or more animals). Scale bar, 50 µm. *p<0.05, **p<0.01 and ***p<0.001.
