# Supplementary material for: Specific targeting of PDGFRβ in the stroma inhibits growth and angiogenesis in tumors with high PDGF-BB expression
Source: Theranostics. 2020 Jan 1;10(3):1122–35. doi: 10.7150/thno.37851 (PMC6956815; doi:10.7150/thno.37851)
Supplement: Supplementary file 1 — Supplementary figures and tables. [file thnov10p1122s1.pdf]

Supplementary information

Title: Specific targeting of PDGFRβ in the stroma inhibits growth and angiogenesis in tumors with high PDGF-BB expression

Authors: Maria Tsioumpekou*[1,2,3], Sara I. Cunha*[3,4], Haisha Ma[3,5], Aive Åhgren[3], Jessica Cedervall[1], Anna-Karin Olsson[1], Carl-Henrik Heldin[1,3] and Johan Lennartsson[2,3]

1. Department of Medical Biochemistry and Microbiology, Uppsala University, Sweden
2. Department of Pharmaceutical Biosciences, Uppsala University, Sweden
3. Ludwig Institute for Cancer Research, Uppsala Branch, Uppsala University, Sweden
4. Department of Immunology, Genetics and Pathology, Uppsala University, Sweden
5. Department of Neuroscience, Uppsala University, Sweden

*These authors contributed equally to the study.

The supplementary file contains:

-Supplementary tables (Table S1-S3)

-Supplementary figures and figure legends (Figures S1-S4)

Gene	Primer (Forward)	Primer (Reverse)
Cspg4	TTGGTCTACACCATCGAGCA	AGGGCTCCTCTGTGTGAGAA
Xiap	CGAGCTGGGTTTCTTTATACCG	GCAATTTGGGGATATTCTCCTGT
Vegf-a	CAGGCTGCTGTAACGATGAA	TATGTGCTGGCTTTGGTGAT

Table S1: List of primer sequences used in this present study.

Antibody	Assay	Dilution	Distributor
**CD31**	IF	1:100	MEC13.3; BD
**podocalyxin**	IF	1:200	AF1556;R&D Systems
**PDGFRβ**	IF	1:100	#3169; Cell Signaling
**α-smooth muscle actin**	IF	1:400	C6198; Sigma
**NG2**	IF	1:200	ab5320; Millipore
**PDGF-BB**	IF	1:100	#07-1437; Millipore
**cleaved caspase 3**	IF	1:400	#9664; Cell Signaling
**phosphorylated Erk1/2**	WB	1:1000	#9101, Cell Signaling
**pS473 Akt**	WB	1:1000	#4060, Cell Signaling
**pY857 PDGFRβ**	WB	1:1000	#3170, Cell Signaling
**NG2**	WB	1:1000	ab5320; Millipore
**PDGFRβ**	WB	1:1000	#3169; Cell Signaling
**α-smooth muscle actin**	WB	1:100	sc-53015; SantaCruz
**Alix**	WB	1:1000	home-made; Lennartsson et al., 2006
**PDGFRα**	WB	1:1000	home-made

Table S2: List of antibodies being used in this present study.

Inhibitors	Final concentration (μM)	Distributor
**1-NaPP1**	1	Taconic Artemis
**Imatinib**	3	Novartis Pharma AG
**CI-1040**	3	Sigma
**AG1296**	10	Calbiochem

Table S3: List of low molecular weight inhibitors used for cell culture experiments in this present study.

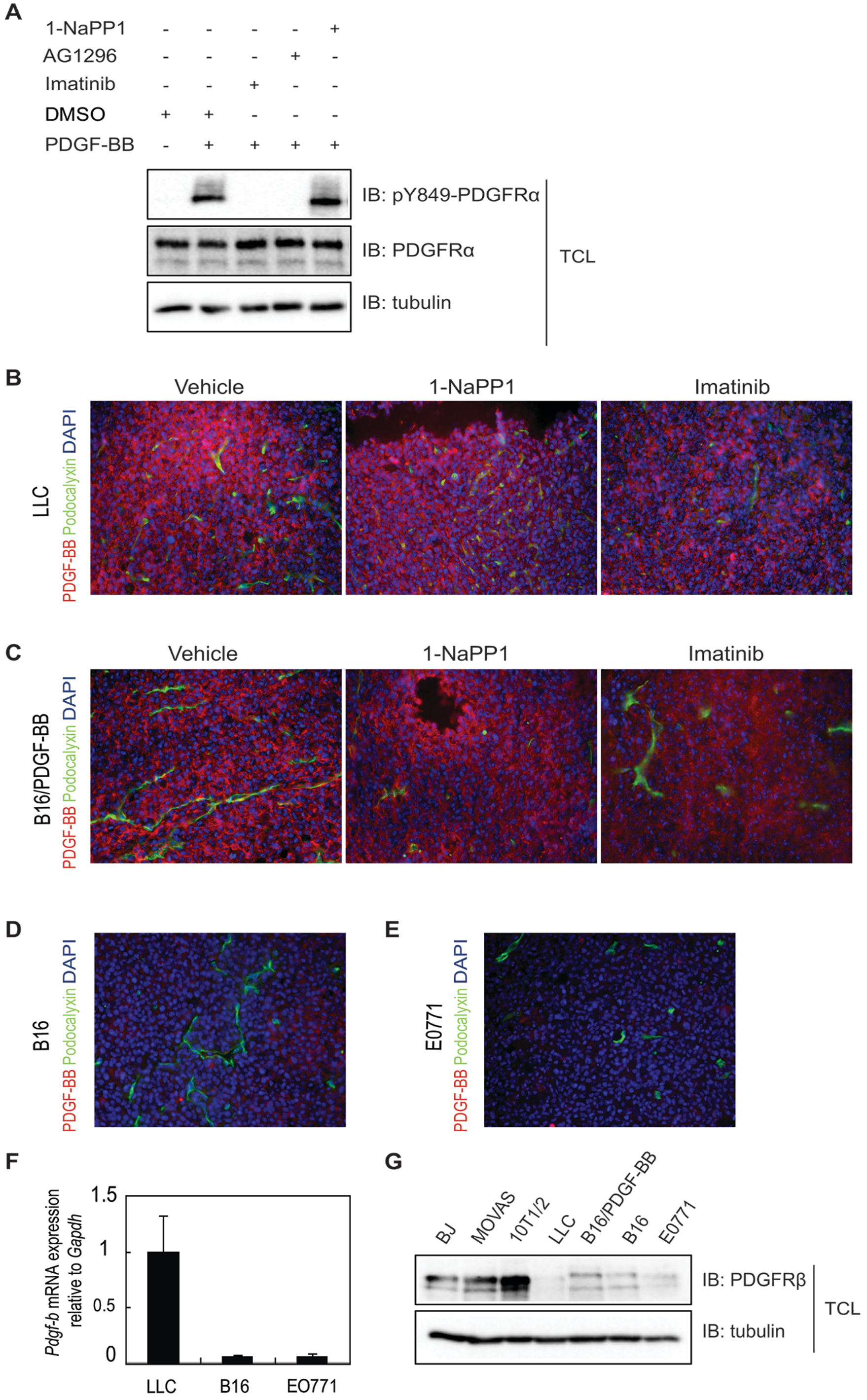

Figure S1: (**A**) 1-NaPP1 does not inhibit the kinase activity of PDGFRα. PAE-PDGFRα were serum-starved overnight, pre-treated with 1-NaPP1, imatinib or another PDGFR inhibitor, AG1296, for 1 h at 37°C, and then stimulated with 20 ng/mL PDGF-BB for 10 min. Total cell lysates (TCL) were collected and PDGFRα kinase activity was evaluated by immunoblotting (IB) using a pY849 PDGFRα antibody. α-tubulin was used as a loading control. (**B-E**) Co-immunostaining of PDGF-BB and podocalyxin in LLC, B16, B16/PDGF-BB and EO771 tumors (PDGF-BB, red; podocalyxin, green; DAPI, blue). (**F**) Analysis of *Pdgf-b* mRNA levels in B16, EO771 and LLC cells by quantitative PCR. Expression levels of *Pdgf-b* were quantified using *Gapdh* as an endogenous loading control. (**G**) Immunoblotting of multiple cell lines for PDGFRβ; IB for α-tubulin was used as a loading control.

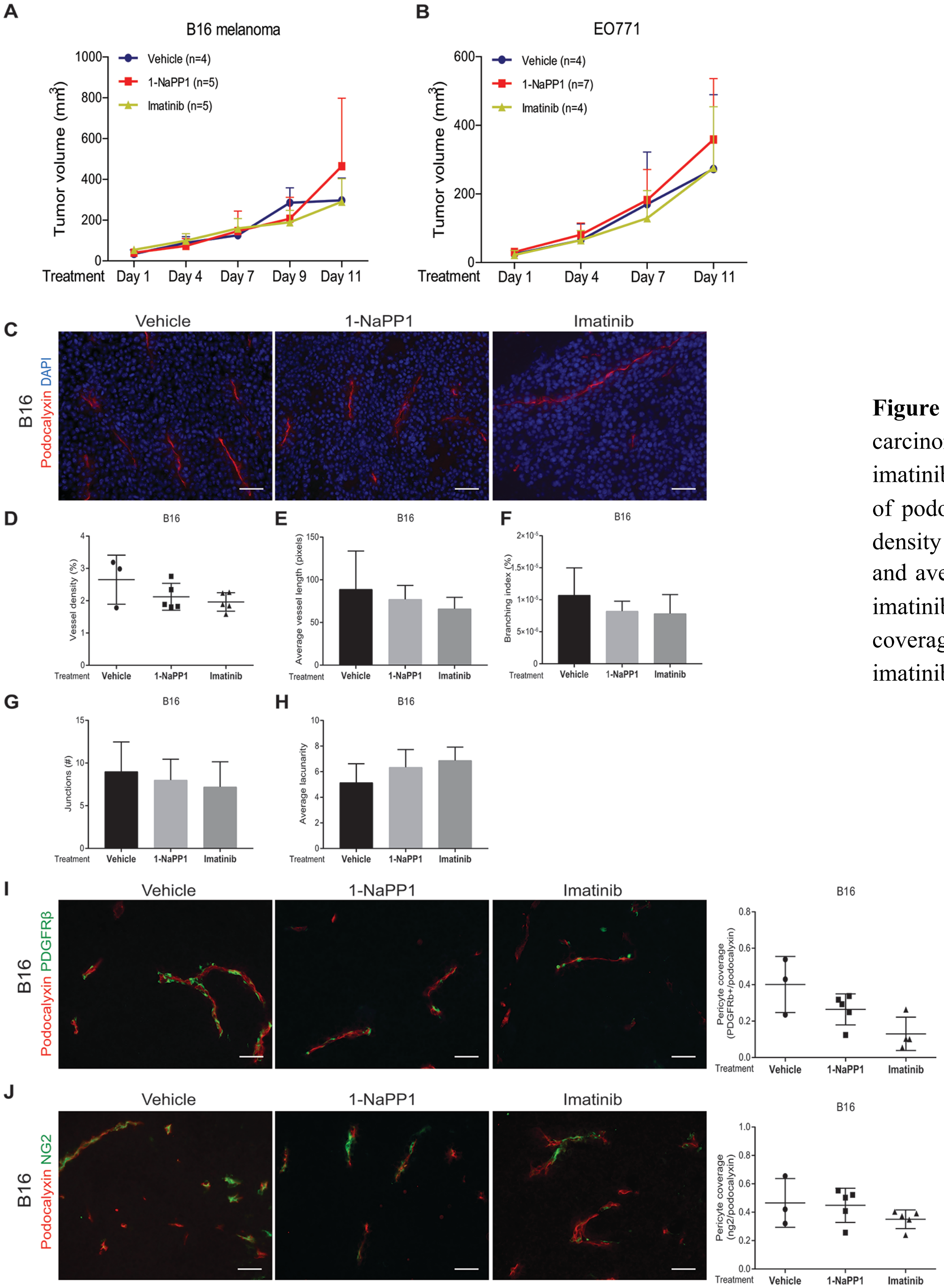

Figure S2: Low PDGF-BB expressing B16 melanoma (A) and EO771 breast carcinoma (**B**) murine tumors are insensitive to treatment with 1-NaPP1 or imatinib in the ASKA mice (n= as stated in the figure). Representative images of podocalyxin-stained vessels (**C**) and analysis of vessel parameters; vessel density (**D**), average vessel length (**E**), branching (**F**), number of junctions (**G**) and average lacunarity (**H**) in B16 tumors treated with vehicle, 1-NaPP1 and imatinib daily for 10 consecutive days. PDGFRβ+ (**I**) and NG2+ (**J**) pericyte coverage quantification in B16 tumors treated with vehicle and 1-NaPP1 and imatinib daily for 10 consecutive days.

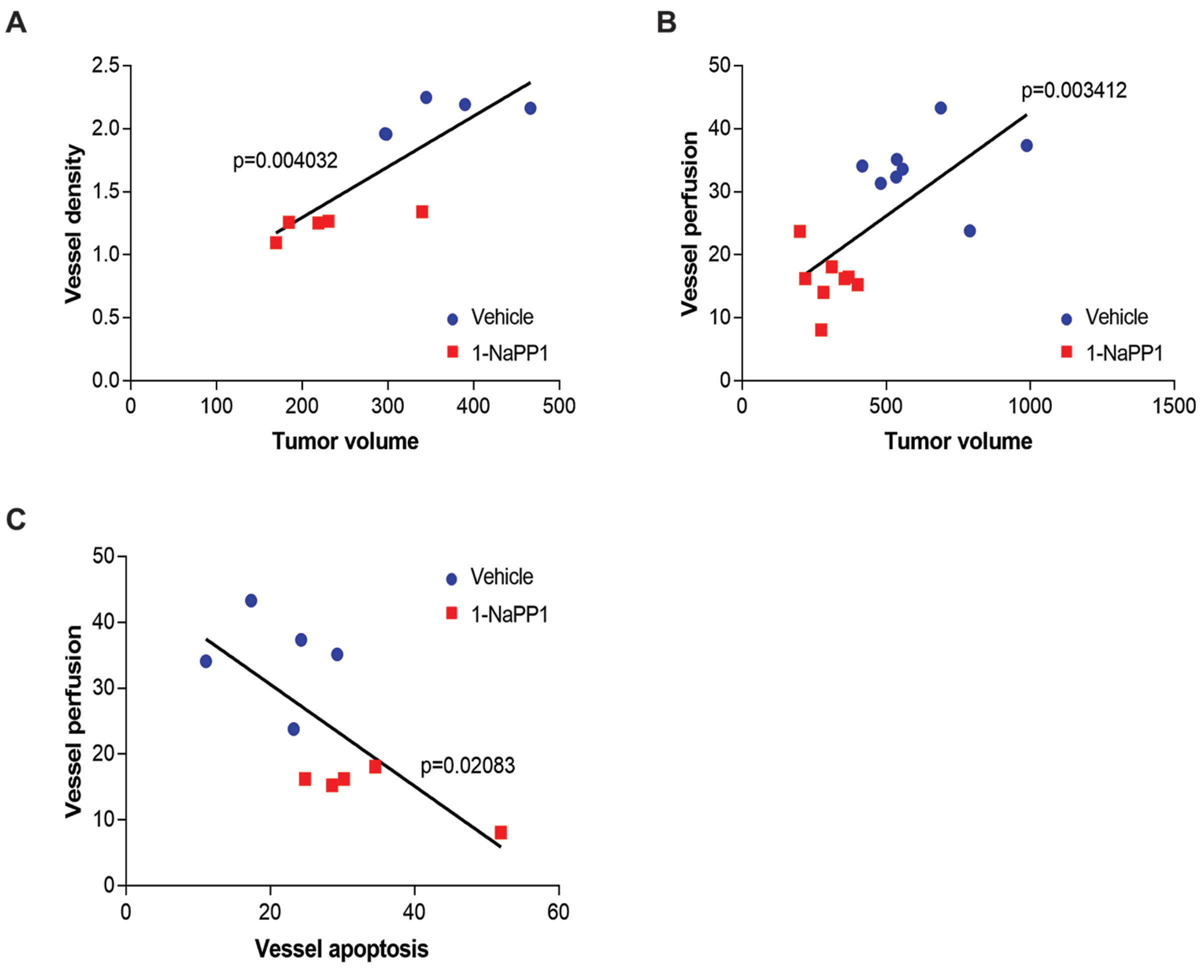

Figure S3: Tumor analysis of control and treated mice after 10 days displayed a positive correlation between tumor volume and vessel density as well as vessel perfusion, as shown from the scatterplots of tumor volume versus vessel density (**A**) and tumor volume versus vessel perfusion (**B**). Scatterplot of vessel perfusion against endothelial cell apoptosis showed a negative correlation (**C**).

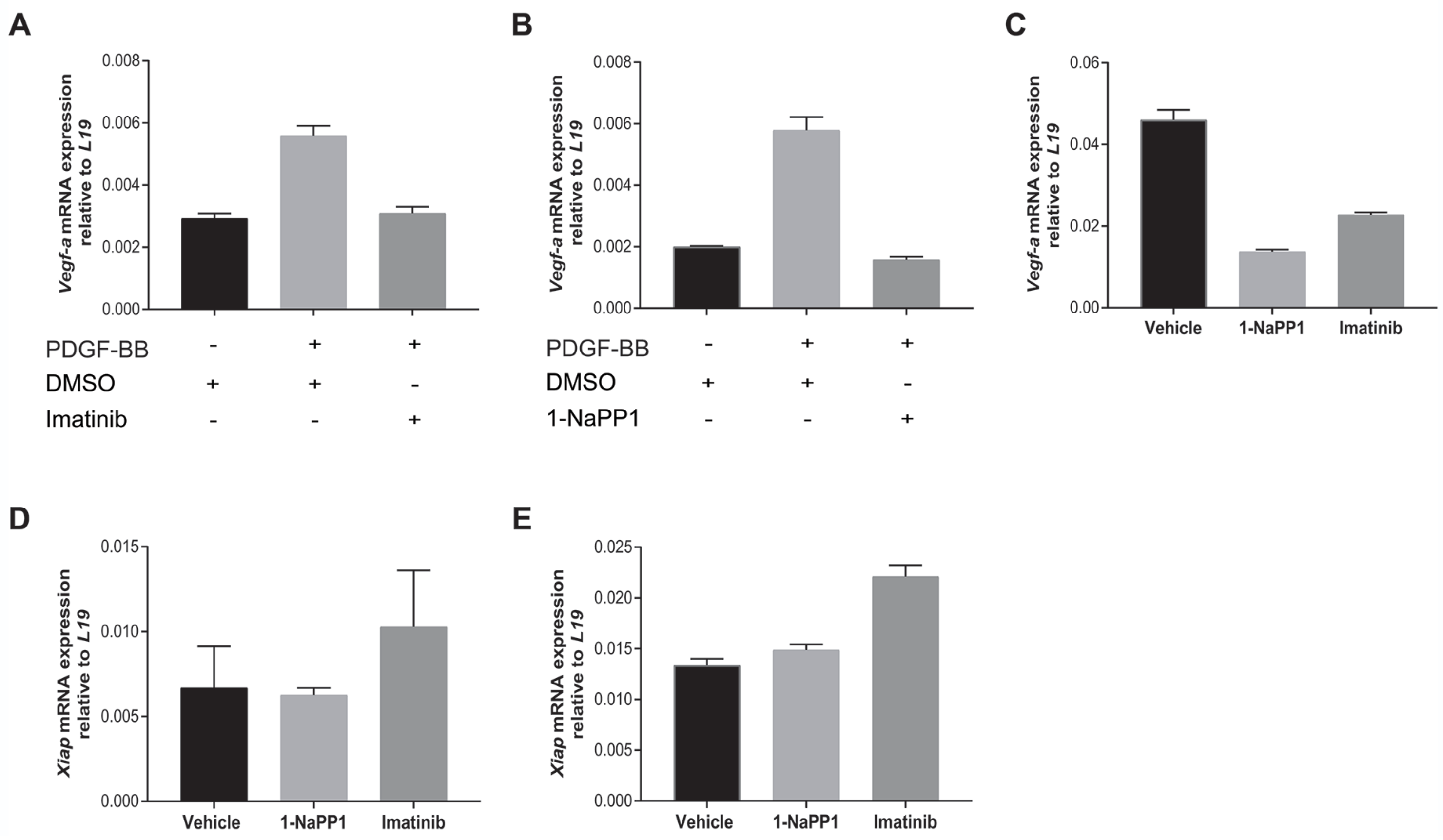

Figure S4: Expression of *Vegf-a* and *Xiap* in cultured cells and tumors treated with 1-NaPP1 or imatinib. To measure *Vegf-a* mRNA expression in 10T1/2 cells (**A**) and ASKA-PDGFRβ MEFs (**B**), serum-starved and treated with vehicle (DMSO), imatinib (3 μM) or 1-NaPP1 (1 μM) in the presence or absence of 20 ng/ml PDGF-BB. After 24 h, mRNA was prepared and quantitative real-time PCR was performed. Error bars indicate standard deviation from triplicate samples. All mRNA expression is relative to *L19* ribosomal gene expression. Two independent experiments were performed. (**C**) PDGFRβ+ cells were isolated from B16/PDGF-BB tumors and *Vegf-a* mRNA expression was measured by qPCR. A representative experiment out of two replicates is shown. To measure *Xiap* mRNA expression in LLC tumors, we performed qPCR of mRNA prepared from isolated PDGFRβ+ cells (**D**) as well as the remaining cell population (**E**). Error bars indicate standard deviation. All mRNA expression is relative to *L19* ribosomal gene expression. Representative data from at least two mice per experimental condition is shown.
